# Efficacy and Safety of Janus Kinase-Inhibitors in Ulcerative Colitis

**DOI:** 10.3390/jcm13237186

**Published:** 2024-11-27

**Authors:** Benedetto Neri, Roberto Mancone, Mariasofia Fiorillo, Sara Concetta Schiavone, Stefano Migliozzi, Livia Biancone

**Affiliations:** Gastroenterology Unit, Department of Systems Medicine, University “Tor Vergata” of Rome, 00133 Rome, Italy; benedettoneri@gmail.com (B.N.); roberto.mancone@yahoo.it (R.M.); fiorillo93@gmail.com (M.F.); saraschiavone27@gmail.com (S.C.S.); stefano.migliozzi@ptvonline.it (S.M.)

**Keywords:** JAK inhibitors, small molecules, Tofacitinib, Filgotinib, Upadacitinib, Ulcerative Colitis, efficacy, safety

## Abstract

**Background:** Janus kinase-inhibitors (JAK-i) have recently been approved for treating patients with Ulcerative Colitis (**UC**); therefore, further information is needed, particularly regarding efficacy and safety. **Objectives:** To provide a comprehensive review regarding the efficacy and safety of currently available JAK-i in UC. **Methods:** The PubMed and Scopus databases were considered, searching for ‘JAK’, ‘JAK-inhibitor’, ‘Janus Kinases’, ‘Tofacitinib’, ‘Filgotinib’, ‘Upadacitinib’, individually or in combination with ‘IBD’, ‘Ulcerative Colitis’, ‘safety’, ‘efficacy’, ‘study’ and ‘trial’. The search was focused on full-text papers published in English, with no publication date restrictions. **Results:** The efficacy and safety of JAK-i approved for treating patients with UC have been summarized. These included Tofacitinib, Filgotinib and Upadacitinib. Findings from both clinical trials and real-life studies in UC were reported, with particular regard to their efficacy in inducing clinical response and remission, steroid-free remission and endoscopic and histological healing. Overall, JAK-i proved to be effective and safe in selected subgroups of patients with UC. The rapid onset of action and the oral route of administration represent the most relevant characteristics of these drugs. Safety concerns using Tofacitinib in subgroups of patients (infections, hypercholesterolemia, venous thromboembolism and cardiovascular events) were initially raised. More recently, all JAK-i for UC showed an overall satisfactory safety profile. However, indication should be carefully given. **Conclusions:** The use of JAK-i UC is promising, although no predictive markers of response are currently available. Optimizing their use, as monotherapy or combined with other immunomodulators, may increase their efficacy in appropriately selected subgroups of patients with UC.

## 1. Introduction

Ulcerative Colitis (UC) is a chronic relapsing Inflammatory Bowel Disease (IBD) characterized by lesions involving the rectum, possibly extending to proximal colonic segments in a continuous fashion [1,2]. The etiology of UC is currently unknown. In terms of pathogenesis, evidence suggests an inappropriate immune response towards luminal antigens, particularly the resident bacterial flora [3]. Genetic susceptibility and environmental triggers have also been shown to be involved in the development of UC [4].

UC onset typically occurs at a young age [5]. The prevalence of UC does not differ in relation to gender, being higher in Western countries (North America and North Europe) [5]. Its incidence has increased worldwide over the last few decades. Probably due to changes in lifestyle and diet, the incidence and prevalence of UC has recently increased also in developing countries (Asia, Middle East and South America) [6].

Inflammatory changes in UC colons are limited to the mucosa and submucosa. An increased number and state of activation of immunocompetent cells are indeed observed in UC colons, thus leading to an increased release of mediators involved in the pathogenesis of the disease. Colonic epithelial cells are also reputed to be involved in the development of chronic inflammation in UC colons [7]. An impaired expression of peroxisome proliferator-activated receptor ɣ (PPAR-ɣ), a nuclear receptor that downregulates inflammation, has been reported in UC [7], together with an increased activation of dendritic cells. These cells express many Toll-like receptors (TLRs), using pattern recognition to activate multiple transcription factors triggering multiple inflammatory cascades. These cascades result in an increased release of cytokines providing prevalent proinflammatory activity. Among these, tumor necrosis factor alpha (TNFα), interleukin (IL)-12 and IL-23 currently represent some of the most relevant therapeutic targets in UC [3]. These cytokines determine the transduction of the proinflammatory message, mediated by intracellular proteins, such as Janus kinase (JAK), which further potentiate lymphocyte activation and proliferation. In several sporadic immune-mediated conditions, subgroups of cytokines have been shown to use JAK-STAT signaling to exert their pathogenic effect [8]. Therefore, during the last few years, efforts have been made to develop treatments able to target the JAK cascade. Recently, small molecules targeting JAK (JAK-inhibitors, JAK-i) indeed showed efficacy for treating diseases (including UC), characterized by a dysregulation of the host immune response [9].

## 2. Therapeutic Targets in UC

Currently, treatment strategies in UC aim to induce and maintain clinical and endoscopic remission [10]. The achievement of clinical remission represents the first target when treating patients with UC. More recently, growing evidence supports the prognostic value of endoscopic remission, as its achievement is associated with a better clinical outcome in the medium and long terms [10,11,12]. Endoscopic remission, defined as mucosal healing (MH), therefore became the main therapeutic target when managing patients with UC, as reported in the recent STRIDE recommendations [10].

Histological remission has been associated with even better long-term positive outcomes, thus supporting this achievement as a possible therapeutic target in UC [10,12,13,14]. Supporting this concept, histological response and remission have been included among secondary outcomes in recent trials using new treatments for UC [15]. Among difficulties related to the use of this treatment target, there is the observation that few scores have been validated for assessing histological activity in UC [12].

## 3. Current Management

Historically, aminosalicylates (5-ASA) are the first choice of treatment for mild-to-moderate UC, while systemic corticosteroids are indicated in moderate-to-severe disease flares [16]. Oral low absorbable corticosteroids with a high first-pass liver metabolism show minimal systemic activity. These include budesonide-multimatrix and beclomethasone dipropionate, both showing efficacy for managing mild-to-moderate UC flares, not responsive to 5-ASA [16,17]. Despite the development of several **effective** immunomodulatory drugs, systemic corticosteroids still remain the mainstay for inducing remission in severe UC [16,17]. In these patients, intravenous corticosteroids should be given (methyl-prednisolone 0.8–1 mg/kg/day), while an equivalent dose of oral prednisone shows efficacy in moderate UC [16,17]. Clinical response should be achieved within two weeks (or 3–5 days in case of acute severe UC, ASUC); then, corticosteroids can be tapered (5 mg/week) [16,17]. Despite the marked efficacy for inducing clinical remission, corticosteroids did not show efficacy for maintaining remission [16,17]. Indeed, to maintain clinical remission, thiopurines and/or biologics should be given [16,17]. Thiopurines include Azathioprine and its active metabolite, 6-Mercaptopurine [16]. Since the 1970s, these drugs have shown significant efficacy for maintaining clinical remission in steroid-dependent moderate-to-severe UC [16]. Since 1995, biologic treatments targeting different inflammatory pathways in UC have been developed, characterized by a relevant efficacy for both inducing and maintaining clinical remission in moderate-to-severe disease [16,17]. The first biologic treatment approved for treating UC is Infliximab (given i.v.), an anti-TNFα monoclonal antibody (anti-TNFα), followed by Adalimumab (given s.c.) [16]. For both treatments, independent studies proved an efficacy significantly higher than placebo for inducing and maintaining clinical remission in UC. Differently from corticosteroids, however, anti-TNFα allows in almost two-thirds of patients with UC the achievement not only of clinical but also of endoscopic remission. In patients with UC, time to relapse has been shown to be longer in the subgroup of patients achieving MH. For these reasons, Infliximab and Adalimumab currently represent key therapeutic agents in UC [18,19]. More recently, Vedolizumab (a gut-selective α4β7 anti-integrin antibody) and Ustekinumab (antibody against the p40 subunit of interleukin-12 and interleukin-23) have been developed, representing additional effective biologics for treating UC [20,21]. Thiopurines and biologics represent treatments able to induce and maintain remission in moderate-to-severe UC. However, only Infliximab can currently be used in patients with ASUC refractory to systemic corticosteroids [16].

## 4. Small Molecules

Small molecules have been recently approved for treating patients with UC [22,23]. Small molecules are characterized by a low molecular weight (<1000 Da). These compounds can diffuse across the cell membrane and target intracellular molecules, thereby regulating cell function. Small molecules are relatively inexpensive, stable and provide improved reproducibility. Moreover, these drugs are characterized by low immunogenicity, thus reducing both the risk of allergic reactions and the loss of response due to neutralizing antibodies. These characteristics, coupled with the possibility of their oral route of administration, account for the success of small molecules for treating UC.

Currently, Tofacitinib, Filgotinib and Upadacitinib are JAK-i approved for UC. Tofacitinib is a pan-JAK-i, mostly targeting JAK1 and JAK3, associated with moderate activity against JAK2 and tyrosine-protein kinase 2 (TYK2). Tofacitinib is the first JAK-i approved for treating UC [16,22,23]. Differently, Filgotinib and Upadacitinib preferentially inhibit JAK1 and have been more recently approved for treating patients with moderate-to-severe UC [16,22,23]. Ozanimod, a sphingosine-1-phosphate receptor (S1P1) inhibitor, is the only other small molecule currently approved for the same indication [16,22,23]. Due to the relevance of this topic and to the quite recent use of small molecules for treating patients with UC, we aimed to provide a comprehensive review regarding the efficacy and safety of currently available JAK-i in UC.

## 5. Materials and Methods

The PubMed and Scopus databases were considered, and the following terms were searched: ‘JAK’, ‘JAK inhibitor’, ‘Janus Kinases’, ‘Tofacitinib’, ‘Filgotinib’, ‘Upadacitinib’, individually or in combination with ‘IBD’, ‘UC’, ‘Ulcerative Colitis’, ‘inhibitors’, ‘safety’, ‘efficacy’, ‘study’ and ‘trial’. The search was focused on full-text papers published in English, and no publication date restrictions were used. Only studies referring to molecules approved for UC treatment were included in the present review. In particular, only findings from systematic review, network meta-analysis, clinical trials and real-world studies reported in PubMed were considered. In order to provide a comprehensive review regarding the approved JAK-i in UC, the efficacy and safety features of these molecules have been reported. In particular, efficacy and safety outcomes from the regulatory trials are summarized in Table 1.

## 6. JAK-Inhibitors in Ulcerative Colitis: Data from Clinical Trials

### 6.1. Tofacitinib

#### 6.1.1. Efficacy

The efficacy of Tofacitinib in inducing clinical remission in moderate-to-severe refractory active UC was first assessed in a phase 2 randomized, placebo-controlled trial. Clinical response at week 8, which was set as primary outcome, was achieved by a higher proportion of patients receiving Tofacitinib 15 mg twice daily (BID) compared to placebo (38/49 [78%] vs. 20/48 [42%]; *p* < 0.01) [24]. As secondary outcomes, clinical remission at 8 weeks occurred in 5/48 (10%) patients receiving placebo compared with 4/31 (13%), receiving 0.5 mg (*p* = 0.76), 11/33 (33%) 3 mg (*p* = 0.01), 16/33 (48%) 10 mg (*p* < 0.001) and 20/49 (41%) 15 mg (*p* < 0.001) of Tofacitinib [24]. Endoscopic activity was assessed at both baseline and at 8 weeks. Endoscopic response was defined as a decrease from baseline in the Mayo endoscopic subscore (≥1 point) and endoscopic remission as a subscore of 0. Endoscopic response was observed in 22 (46%) patients on placebo versus 16 (52%) receiving 5 mg (*p* = 0.64), 19 (58%) 3 mg (*p* = 0.30), 22 (67%) 10 mg (*p* = 0.07) and 38 (78%) 15 mg of Tofacitinib (*p* = 0.001) [24]. Endoscopic remission at week 8 occurred only in one (2%) patient receiving placebo. When compared to placebo, a higher proportion of patients receiving 3 mg (6/33 [18%]; *p* = 0.01), 10 mg (10/33 [30%]; *p* < 0.001) and 15 mg of Tofacitinib (13/49 [27%]; *p* < 0.001) showed endoscopic remission at week 8 [24].

On the basis of the above reported findings, two randomized, double-blind, placebo-controlled trials (OCTAVE 1–2) were made in order to confirm the efficacy of this compound [25]. In these induction trials, clinical remission at week 8 was set as the primary outcome [25]. In these trials, a significantly higher proportion of patients with UC treated with Tofacitinib versus placebo was observed (OCTAVE Inductions 1 and 2: 18.5% vs. 8.2%, *p* = 0.007 and 16.6% vs. 3.6%, *p* < 0.001, respectively) [25]. Interestingly, a rapid onset of action using Tofacitinib was reported, as after 3 days of treatment a significant reduction in UC-related symptoms and in rectal bleeding was observed [25]. In the OCTAVE Inductions 1 and 2 trials, MH at 8 weeks occurred in a significantly higher proportion of patients in the 10 mg Tofacitinib group than in the placebo group (Table 1). This finding was comparable between TNFα-antagonist experienced and naïve patients.

The efficacy in terms of maintenance of the remission of Tofacitinib was assessed in the OCTAVE Sustain trial [25]. In this trial, dose-dependent efficacy of Tofacitinib was observed [25]. At 52 weeks, clinical remission was indeed observed in 34.3% of patients with UC treated with 5 mg Tofacitinib vs. 40.6% of those treated with 10 mg and 11.1% of patients on placebo (*p* < 0.001 for both comparisons) [25]. Tofacitinib treatment was also associated with the achievement of endoscopic remission observed in a high proportion of patients with UC, defined as Mayo endoscopic subscore 0–1 [25]. At 52 weeks, MH was indeed observed in a significantly higher proportion of patients with UC treated with 5 mg (37.4%) or 10 mg Tofacitinib (45.7%) when compared to placebo (13.1%; *p* < 0.001 for both) [25].

#### 6.1.2. Safety

##### Thromboembolic Events

In the OCTAVE Induction trials [25], the reported safety profile was satisfactory at both 5 mg and 10 mg doses. Thereafter, several meta-analyses supported a comparable safety of Tofacitinib and biologics [26]. However, initial data in patients with rheumatoid arthritis (RA) raised concerns about the potential risk of venous thromboembolism (VTE) for Tofacitinib [27]. Indeed, in July 2019, a “black box warning” was placed on Tofacitinib in patients with RA regarding a possible associated risk of pulmonary embolism [28]. In these patients, the greatest thrombotic risk was observed when comparing Tofacitinib 10 mg with anti-TNFα (19/3884 patients–year vs. 3/3982 patients–year, respectively) [29]. In particular, VTE risk was significantly higher in patients with a previous history of malignancy, age > 50 years and with ≥ one cardiovascular risk factor [29]. In UC, a higher VTE risk was reported in the subgroup of patients treated in the long term with Tofacitinib 10 mg b.i.d. and previous risk factors for thromboembolic events [26,27]. Thus, maintenance doses of 10 mg b.i.d. are currently not recommended in patients with UC with known risk factors for VTE, unless no alternative treatment is available [16,17]. As observed in patients with RA, patients with active IBD have consistently been shown to bear a higher risk of thromboembolic events [30,31]. On the basis of these observations, the real role played by Tofacitinib in further raising this risk of VTE in patients with IBD, tailored in a patient’s basis, needs to be further investigated. Findings from the OCTAVE 1 and 2 trials suggest that VTE events in treated patients are rare, and real-world safety signals are comparable to those reported in clinical trials [25].

##### Infections

Tofacitinib use has been associated with a higher risk of Herpes Zoster virus (HZV) infection, even though a mild course has been reported in most cases [25,26]. While for anti-TNFα treatments, tuberculosis risk is a relevant issue, in the OCTAVE trials no cases of tuberculosis have been reported using Tofacitinib [24,25]. Even though few cases of tuberculosis have been reported in RA, in UC, this risk seems to be low and thus of concern only in endemic areas [27].

##### Cancer Risk

Among potential AEs related to the use of the currently approved small molecules for treating patients with UC, concern regards the cancer risk. The short-term follow-up of patients enrolled in clinical trials significantly weakens the relevance of current findings regarding the cancer risk associated with the use of this drug in patients with UC.

In 2017, the efficacy and safety of Tofacitinib in UC were assessed in a large population of patients in three phase 3, randomized, double-blind, placebo-controlled trials (OCTAVE Inductions 1 and 2 and OCTAVE Sustain, including 598, 541 and 593 patients, respectively). In these three trials, non-melanotic skin cancers (NMSCs) occurred in five patients treated with Tofacitinib versus one on placebo, while cardiovascular events in five patients versus none, respectively. However, Tofacitinib was associated with increased lipid levels [24,25]. In 2021, findings from the Tofacitinib UC clinical development program reported infrequent malignancies, at rates comparable with those reported in RA and psoriasis, or using biologics in UC [32]. Risk factors for malignancies (excluding NMSC) were UC duration (1.07 [1.02–1.11]), age (1.05 [1.01–1.08]), prior NMSC (5.09 [1.18–21.96]) and BMI (1.07 [1.01–1.14]) [32]. Subsequently, in 2022, safety data were analyzed from three randomized, placebo-controlled studies (OCTAVE Inductions 1 and 2; OCTAVE Sustain) and an open-label study (OCTAVE Open) ongoing at the time of the study. Overall, history of NMSC (9.09; *p* = 0.0001), anti-TNFα failure (3.32; *p* = 0.0363) and age (HR per 10-year increase 2.03; *p* = 0.0004) were identified as independent risk factors for NMSC [33].

Reassuring findings derive from very recent findings from a summary of Tofacitinib safety (9.2 years maximum exposure). UC population includes patients completing phase 2/3 placebo-controlled studies, a randomized phase 3b/4 study and an open-label, long-term extension study [34]. The study reports data from 1157 patients with UC treated with ≥1 Tofacitinib dose (5 or 10 mg b.i.d.) for a median of 1.7 [0.0–9.2] years. AEs were observed in 994 (85.9%) and severe AEs (SAEs) in 254 (22.0%) patients with UC. Incident risk (IR) using Tofacitinib (5 or 10 mg) was associated > 1 case/100 PY only for (IRs [95% CI]): serious infections (1.80 [1.37–2.32]) and HZ (nonserious and serious) (3.24 [2.63–3.94]). Differently, IRs for AEs were <1 case/100 PY for the other outcomes considered (deep venous thrombosis 0.06 [0.01–0.22], pulmonary embolism 0.18 [0.07–0.40], deaths, malignancies excluding or not NMSC, major adverse cardiovascular events, opportunistic infections and gastrointestinal perforations).

Despite recent reassuring data regarding the safety of Tofacitinib, as for all recently developed treatments, additional information regarding the safety in the long term in real life is required. The only systematic review and meta-analysis using JAK-i for RA, psoriasis, ankylosing spondylitis and IBD reported a comparable risk of developing either NMSC or any cancer excluding NMSC [35], as stated by current ECCO guidelines [36].

##### Pregnancy

The active metabolite of JAK-i, as also for other small molecules, can cross the placenta during the first trimester [37], thus raising concern about the safety of this treatment during pregnancy. Preclinical studies using Tofacitinib showed that exposure to doses much higher than the therapeutic dose can cause fetal malformations [38]. At therapeutic dose, no fetal deaths were reported. Embryotoxicity and teratogenicity at higher doses than those administered in humans were observed in animal models [39]. Current data regarding women exposed to JAK-i during pregnancy are limited. Indeed, only for Tofacitinib, these data are available. In a 2018 study involving 45 women with UC exposed to Tofacitinib, the incidences of spontaneous abortions and malformations were 10.7% and 3.6%, respectively [39,40]. This finding suggested that fetal malformations and spontaneous abortion risks are comparable to the risk observed in the general population [39,40]. However, current indications include the discontinuation of Tofacitinib and Upadacitinib at least 4 weeks before conception and during lactation, while a 1-week wash-out is recommended for Filgotinib [41,42,43].

##### Effects on Lipid Metabolism

High serum lipid levels are known risk factors for major cardiovascular AE. In UC trials using Tofacitinib [25], Low-Density Lipoprotein (LDL) and High-Density Lipoprotein (HDL) cholesterol levels increased after 8 weeks of treatment, normalizing after treatment discontinuation and associated with stable total-to-HDL cholesterol ratio. Taking into account the reported VTE risk in Tofacitinib-treated patients, the dose-dependent serum lipid increase appeared as a relevant issue. Therefore, based on these findings, current indications indicate the need to monitor serum lipid levels within 2 months since treatment. However, long-term Tofacitinib use has been reported not to determine significant changes in terms of lipid profile and overall, when reported, not to increase the risk for major cardiovascular AEs [25,30]. In a recent meta-analysis, all the JAK-i approved for RA were reported to determine a mean increase of 8.11 mg/dL of HDL and of 11.37 mg/dL LDL serum levels when compared to baseline [44].

### 6.2. Filgotinib

#### 6.2.1. Efficacy

Filgotinib is one of the two selective JAK-i currently approved for UC treatment, preferentially inhibiting JAK1 over JAK2, JAK3 and tyrosine kinase 2. Filgotinib has been evaluated in several randomized controlled trials in patients with RA [45], psoriatic arthritis [46] and ankylosing spondylitis [47]. In CD patients, Filgotinib 200 mg was superior to placebo for inducing clinical remission in the phase 2 FITZROY trial [48]. Efficacy and safety of Filgotinib in UC were assessed in a phase 2b/3 double-blind, randomized, placebo-controlled trial [49]. The primary end-point was clinical remission defined as Mayo endoscopic subscore of 0 or 1, rectal bleeding subscore of 0 and ≥1 point decrease in stool frequency from baseline for a subscore of 0 or 1. In this trial, endoscopic remission was defined as a Mayo endoscopic subscore of 0 [50], while histologic remission was based according to the Geboes scale [51]. Patients were randomized to receive placebo or Filgotinib (100 mg or 200 mg), subgrouped according to previous exposure to anti-TNFα. At week 10, a greater proportion of patients given Filgotinib 200 mg reached clinical remission than those given placebo (induction study A: 26.1% vs. 15.3%, *p* = 0.0157; induction study B: 11.5% vs. 4.2%, *p* = 0.0103). In the maintenance phase, at week 58, clinical remission was observed in 37.2% of patients given Filgotinib 200 mg versus 11.2% in the respective placebo group (*p* < 0.0001). The proportion of patients reaching clinical remission was not significantly different between Filgotinib 100 mg and placebo at week 10, but it was significantly higher by week 58 (23.8% vs. 13.5%, *p* = 0.0420) [49].

In the induction trial, in biologic naïve patients, a higher frequency of endoscopic healing was reported in patients assuming Filgotinib 200 mg vs. placebo (3.6% vs. 12.2%, *p* = 0.0047) [49]. Differently, the frequency of MH was comparable between placebo and Filgotinib (at both 100 and 200 mg dosages) in patients previously treated with biologics. In both biologic naïve and experienced patients, a higher frequency of histological healing was observed in those receiving Filgotinib 200 mg when compared to those receiving placebo (16.1% vs. 35.1%; *p* < 0.0001 and 8.5% vs. 19.8%; *p* = 0.0019, respectively) [49].

In the maintenance study, a greater proportion of patients in the Filgotinib 200 mg group achieved MH and histological remission than in the placebo group (6.1% vs. 15.6%; *p* = 0.0157 and 13.3% vs. 38.2%; *p* < 0.0001, respectively), while the same efficacy was not statistically significant for the Filgotinib 100 mg group [49].

#### 6.2.2. Safety

In patients treated with Filgotinib for diseases other than UC, a favorable safety profile of this drug was reported [52,53]. Indeed, the reported AEs were mostly mild or moderate in terms of severity [52,53]. In accordance with data from trials including patients treated for diseases other than UC, the AE rate in the three UC trials was relatively high, although mild to moderate in terms of severity and comparable to that of placebo [49]. The distribution of treatment-emergent AEs during the induction studies was similar in all groups (placebo: 56.3%, Filgotinib 100 mg: 50.4%, Filgotinib 200 mg: 56.3%) [49]. The most frequent AEs during the induction studies were nasopharyngitis, headache and UC worsening; while, in the maintenance study, arthralgia, abdominal pain and upper respiratory tract infections were also reported [49].

#### 6.2.3. Thromboembolic Events

One patient in the Filgotinib 200 mg arm with a medical history of hypothyroidism, idiopathic pulmonary symptoms and assuming prednisone had a pulmonary embolism during the induction study [49]. Neither venous thrombosis nor pulmonary embolism occurred in patients receiving Filgotinib in the maintenance study [49]. Data from patients using Filgotinib for other diseases suggest that VTE should not be a relevant issue related to this drug [45,46,48,53].

#### 6.2.4. Infections

HZV infection was reported in a minority of patients during the induction and maintenance study [49]. HZV infections were also observed in trials of Filgotinib in patients with RA; however, they were mild and limited to the dermatome [54]. In UC, one patient receiving Filgotinib 200 mg during the induction study developed a successfully treated opportunistic infection (esophageal candidiasis) [49]. Data from other diseases suggest a comparable opportunistic infection rate between placebo and Filgotinib [45,46,48,53].

#### 6.2.5. Cancer Risk

No malignancies were reported in the Filgotinib induction and maintenance trials in UC [49]. Overall, JAK-i administered in patients for all indications were associated with a higher incidence of malignancy compared with TNFα inhibitors, but not placebo or methotrexate, representing rare events in all comparisons [55]. However, data regarding specifically Filgotinib and in particularly long-term data are currently lacking.

#### 6.2.6. Pregnancy

As for Filgotinib, embryotoxicity and teratogenicity (causing malformations of central nervous, musculoskeletal, respiratory and cardiovascular systems) were demonstrated in rats and rabbits at exposures comparable to the 200 mg daily dosing regimen in humans [43]. The 2022 ECCO guidelines therefore contraindicate the use of Tofacitinib and Filgotinib during pregnancy [56].

#### 6.2.7. Testicular Toxicity

Two safety studies examining the effect of Filgotinib on sperm parameters were conducted, due to safety concerns regarding testicular toxicity. They were conducted in patients with IBD (MANTA, NCT03201445) or RA and other inflammatory arthropathies (MANTA-Ray, NCT03926195) [57,58]. Results of both studies concluded that Filgotinib treatment is not associated with negative effects on testicular function.

### 6.3. Upadacitinib

#### 6.3.1. Efficacy

Upadacitinib [28] is the other selective JAK-i currently approved for the treatment of UC, engineered to have greater inhibitory effects for JAK1 than JAK2, JAK3 and tyrosine kinase 2. In a phase 2b (U-ACHIEVE substudy 1), dose-ranging, placebo-controlled induction study, patients with moderate-to-severe UC received Upadacitinib (doses: 7.5 mg, 15 mg, 30 mg and 45 mg once daily) [59]. Upadacitinib 45 mg once daily showed an optimal benefit-risk profile when compared with lower doses and was therefore selected for the phase 3 as induction dose [59]. However, for the phase 3 maintenance doses, two doses (15 mg and 30 mg once daily) lower than the induction dose were chosen, in relation to potential more favorable long-term safety profile [60]. Both doses also showed higher efficacy than placebo in the phase 2b study [59]. In the phase 3 trial, the primary end-point was clinical remission, defined as adapted Mayo score ≤ 2, with stool frequency subscore ≤ 1 and not greater than baseline, rectal bleeding subscore 0 and endoscopic subscore ≤ 1 without friability [60]. Endoscopic activity was assessed using the endoscopic Mayo score and histological activity using the Geboes score [51]. Patients were subgrouped according to the biologic exposure.

Clinical remission was achieved by a significantly higher proportion of patients in the Upadacitinib 45 mg group (83 [26%] of 319 patients in UC1 and 114 [34%] of 341 patients in UC2) than in the placebo group (7/154 [5%] patients in UC1 and 7/174 [4%] patients in UC2; *p* < 0.0001) [60]. In the maintenance stud, a significantly higher frequency of clinical remission was observed in patients receiving Upadacitinib vs. placebo (15 mg: 63/148 [42%]; 30 mg: 80/154 [52%]; placebo: 18/149; *p* < 0.0001) [60].

Endoscopic remission was observed in the induction study in a higher proportion of patients treated with Upadacitinib than placebo (2 [1%] vs. 44 [14%]; *p* < 0.0001 and 3 [2%] vs. 62 [18%]; *p* < 0.0001). In the maintenance study, patients on Upadacitinib had both 15 and 30 mg dosages (8 [6%] vs. 36 [24%]; *p* < 0.0001 and vs. 40 [26%]; *p* < 0.0001, respectively) [60].

Accordingly, histologic improvement was also reached by a significantly higher proportion of patients treated with Upadacitinib than placebo in both the induction studies (35 [23%] vs. 175 [55%]; *p* < 0.0001 and 43 [5%] vs. 212 [62%]; *p* < 0.0001) [60]. Moreover, in both induction and maintenance studies, combined endoscopic–histologic improvement and healing were observed significantly more frequently in patients treated with Upadacitinib vs. placebo [60].

#### 6.3.2. Safety

In terms of safety profile, the use of Upadacitinib, as other selective JAK-i, currently appears not to be associated with SAEs [35,61,62]. In particular, in one of the two induction studies, the proportion of patients with reported AEs was similar in the Upadacitinib 45 mg and placebo groups (180/319 [56%] vs. 93/155 [60%]). Differently, in the other induction study, the frequency of AEs was higher in the Upadacitinib 45 mg group than in the placebo group (182/344 [53%] vs. 70/177 [40%]) [60]. However, in both induction studies, serious AEs and AEs leading to treatment discontinuation were less frequent in the Upadacitinib 45 mg group than in the placebo group [60]. In the maintenance study, AEs were comparable between patients on Upadacitinib 30 and 15 mg and placebo. Overall, adverse events of special interest were infrequent in all studies [59,60].

#### 6.3.3. Thromboembolic Events

Across the Upadacitinib rheumatologic trials, six VTEs were reported over 461 treatment-arm patient years, compared with one event in 366 placebo-arm patient years [63]. No thrombotic events have been reported using Upadacitinib in RA, but the drug was only recently in the market (in 2019), and long-term studies in larger populations are warranted [64]. In UC trials using Upadacitinib, no cases of VTE were reported during the induction trials and two in patients using Upadacitinib 30 mg, with no difference in terms of frequency when compared with placebo [59,60].

#### 6.3.4. Infections

The risk of HZV has been reported for all JAK-i that are either available or under development. For Upadacitinib, three cases were reported during the UC trials [60].

#### 6.3.5. Cancer Risk

When considering trials using Upadacitinib, cases of any malignancy occurred in only one patient in the induction and in two patients in the maintenance trial [60]. Moreover, in long-term studies, comparable malignancy rates were observed using Upadacitinib and Filgotinib in RA patients versus the overall population of RA patients [40].

#### 6.3.6. Pregnancy

Current indications include the discontinuation of Tofacitinib and Upadacitinib at least 4 weeks before conception. The 2022 ECCO guidelines contraindicate the use of Tofacitinib and Filgotinib during pregnancy [56]. Similar recommendations can also be made for Upadacitinib.

## 7. JAK-Inhibitors in Ulcerative Colitis: Data from Real-Life Studies

### 7.1. Tofacitinib

Real-life observations are needed in order to confirm findings from clinical trials. Due to their relatively recent introduction, the effectiveness of JAK-i in UC in real-life studies is sparse and based on a relatively limited number of patients.

In 2022, a systematic review and meta-analysis including 1162 patients with UC treated with Tofacitinib reported, among other outcomes, the rate of clinical remission, clinical response and MH [65]. Clinical remission, assessed in 13 studies (including 755 patients), was reported in 35%, 47% and 38.3% of patients at weeks 8, 12–16 and at 6 months, respectively. Moreover, clinical response was reached in 62.1%, 64.2%, 50.8% and 41.8% of patients at weeks 8 and 12–16 and at 6 and 12 months, respectively. MH, evaluated in seven studies at weeks 8 and 12–16, was achieved in 48.3% and 45.3% of patients, respectively [65].

In an Indian population of biological naïve patients with UC, clinical remission after the eight-week induction period was reported in 70.2% of patients [66]. In a South Italian population, similar rates of clinical response (74.8%) were reported at 8 weeks [67]. In a retrospective single-center observational analysis of 111 patients with UC treated with Tofacitinib, the clinical response and remission rates were 66.3% and 50.5% (week 8) and 47.1% and 43.5% (week 48), respectively. Responsiveness was reported not to be related to previous anti-TNFα exposure (*p* = 0.25). At week 8, endoscopic score of activity (Mayo score) was significantly lower in responders vs. non-responders (OR 0.61 [0.45–0.82]; *p* = 0.001) [68].

Regarding histological healing, this issue was assessed in a recent retrospective multicentric cohort study including 42 patients with UC treated with Tofacitinib (previous exposure to anti-TNFα in 93% and to Vedolizumab 81%). Findings showed histological remission according to the Nancy index in 19% of patients and a reduction in the epithelial neutrophilic infiltrate in 24%, respectively [15].

The most recent retrospective, multicenter study on safety and efficacy of Tofacitinib in UC included 391 patients (median endoscopic Mayo score of three). Among these patients, 11.8% were biologic naïve, while 84% were previously exposed to anti-TNFα and 64% to Vedolizumab). Half of these patients were using corticosteroids at enrollment, and steroid-free remission was observed in 24% and 41% of patients at 12 and 52 weeks, respectively. Endoscopic remission was reached by 3% of patients (*p* < 0.001). AEs were observed in 18% of patients, including SAEs in 3.1% of patients. In half of the cases, Tofacitinib discontinuation was related to severe infection (two HZV, one pneumonia, one perianal abscess, one carbuncle and one Clostridioides difficile infection). In the remaining half of cases, Tofacitinib was discontinued due to dyspnea, vertigo, nausea and gastric pain [69].

Real-life studies are also useful in order to search for potential clinical predictive markers of response. A higher rate of clinical remission has been reported in patients with UC treated with Tofacitinib used as a first-/second-line rather than a third-/fourth-line treatment (*p* = 0.007) [70]. 

### 7.2. Filgotinib

The efficacy of Filgotinib in UC in a real-life setting has currently been assessed in study populations even smaller than those of Tofacitinib. Indeed, this issue was investigated in a retrospective study including 91 patients with UC (67% both biologic and small-molecule naïve), failing one or more advanced therapies (20.9% and 12.1%, respectively) [71]. In this study population, clinical remission was achieved in 71.9% at 12 weeks and in 76.4% of patients at 24 weeks [71].

In a different retrospective study, lower rates of clinical remission were reported in 25 patients with UC treated with Filgotinib. This study included 36% biologic and small-molecule naïve patients and 52% biologic and small-molecule multi-failure patients. Most of these patients with UC showed moderate endoscopic activity. Clinical remission was observed in 52% of patients at 24 weeks, while endoscopic remission was achieved in 47% of patients [72].

### 7.3. Upadacitinib

Among real-life studies investigating the effectiveness of Upadacitinib in UC, a single-center, prospective analysis was performed in 44 patients with UC followed up for 8 weeks after treatment. All of these patients were previously exposed to biologics, most of them to more than one advanced therapy. After four-week treatment, clinical response was observed in 76% of patients and clinical remission in 81.5%. At week 8, clinical response and remission were observed in 85.2% and 81.5% of patients, respectively. In patients previously exposed to Tofacitinib, clinical remission at week 8 was observed in 77.8% of patients [73].

In a retrospective study, including 75 patients with UC treated with Upadacitinib (with previous use of Tofacitinib in 30%), clinical remission was reported in 64% of patients between weeks 8 and 16 [74]. In a subgroup of these patients (26 out of 75), rates of endoscopic response and remission after a median of 23 weeks were observed in 61.5% and 34.6% of patients, respectively. No differences in terms of clinical remission were observed in Tofacitinib treated vs. naïve patients (*p* = 0.3) [74].

Upadacitinib use after other JAK-i failure was assessed in a single-center case series including 16 patients with UC who received Upadacitinib after failing Tofacitinib. Among these 16 patients, 5 achieved steroid-free clinical remission, 5 endoscopic response and 2 patients achieved endoscopic remission [75]. In another case series, among six patients with UC who presented an inadequate response or intolerance to Tofacitinib or Filgotinib, five (83.3%) patients were reported to achieve clinical remission after 2 months of treatment with Upadacitinib [76].

Limited head-to-head real-life studies have also been reported. A retrospective multicenter cohort study compared the efficacy of Ustekinumab and Upadacitinib in a cohort of 218 patients with UC (70 Upadacitinib, 148 Ustekinumab). When comparing these two groups, the number of patients treated with Vedolizumab and ≥1 anti-TNFα was higher in the Ustekinumab group. Differently, in the Upadacitinib group, a more severe clinical activity, as assessed by the Simple Clinical Colitis Activity Index (SCCAI), was observed. Moreover, all patients had a history of previous exposure to biologics and of failure to advanced therapies. Tofacitinib exposure and endoscopic activity were comparable between the two treatment groups. In the analysis, significantly higher odds of clinical response and steroid-free clinical remission at 8 to 16 weeks, as also of endoscopic remission within 52 weeks, were observed in the subgroup of patients treated with Upadacitinib vs. Ustekinumab [77].

A retrospective cohort study compared the effectiveness of Upadacitinib versus Tofacitinib in 526 patients with UC treated with Upadacitinib, matched with 1.149 Tofacitinib-treated patients. Matching was made according to several variables: demographics, comorbidities, prior UC medications (including recent oral or intravenous corticosteroids), hemoglobin levels, C-reactive protein, albumin and calprotectin. No significant differences were observed between the two groups in terms of need for intravenous corticosteroids and/or colectomy within 6 months (aOR 0.75 [0.49–1.09]). Differently, lower rates of colectomy at 12 months were reported in the Upadacitinib group [78].

Another head-to-head retrospective cohort study was conducted to compare effectiveness of Upadacitinib and Tofacitinib. In the enrolled population of 155 patients, 81 patients were treated with Upadacitinib (24 with prior Tofacitinib exposure) and 74 with Tofacitinib. Upadacitinib was associated with significantly higher odds of steroid-free clinical remission at 52 weeks (OR 3.01 [1.39–6.55]) versus Tofacitinib. Differently, no differences were reported in terms of endoscopic response/remission [79].

## 8. JAK-i in Acute Severe Ulcerative Colitis

ASUC is a well-defined clinical entity, occurring at least once in 20–25% of patients with UC. The standard of care is i.v. corticosteroids followed by Infliximab or Cyclosporine when corticosteroid treatment fails. Very limited data are provided about the use of JAK-i in this clinical setting, but these treatments represent a promising therapeutic option due to their efficacy and rapid onset of action.

Recently, the first single-center double-blind, placebo-controlled trial on the use of Tofacitinib in the ASUC setting has been published [80]. The primary outcome of the trial was to evaluate the rate of clinical response (defined as a reduction in the Lichtiger score by >3 points and an absolute score < 10 for two consecutive days without the need for rescue therapy) at day 7 when adding Tofacitinib to i.v. corticosteroids. Higher rates of response were reported in patients on Tofacitinib plus steroids versus steroids plus placebo (83.01% vs. 58.82%; *p* = 0.007). The need of rescue therapy at day 90 was also reduced in the subgroup of patients assuming Tofacitinib (*p* = 0.003). The majority of AEs were mild, with one patient in the Tofacitinib group developing dural venous sinus thrombosis [80].

In a recent interim analysis in a Canadian phase 4, prospective interventional trial of Tofacitinib in ASUC (TRIUMPH), 24 patients with ASUC received Tofacitinib 10 mg bid [81]. At day 7, clinical response was achieved in 14 (58.3%) patients with a mean time to clinical response of 2.4 days. Overall, endoscopic improvement (Mayo endoscopic subscore of 0–1) assessed at month 6 was observed in 8/24 (33.3%) patients. One patient developed an SAE (stroke, after 3 days of Tofacitinib) [81].

A systematic review analyzed 21 studies including 148 cases regarding patients with UC treated with Tofacitinib as second-line treatment after failing steroids in previous Infliximab failures or as third-line after sequential steroid and Infliximab or Cyclosporine failure. Overall, despite incomplete data, 85%, 86% and 69% colectomy-free survival was reported at 30, 90 and 180 days, respectively. Clinical remission and endoscopic remission were achieved in 35–69% and in 55% of patients, respectively. AEs occurred in 22 patients, mainly represented by infectious complications other than HZV (*n* = 13), requiring Tofacitinib discontinuation in seven patients [82].

Few pieces of data are reported about the use of Upadacitinib in the ASUC setting. A case series of four hospitalized patients with ASUC, not responding to i.v. steroids and Infliximab, received, off-label, 45 mg/day Upadacitinib. Clinical improvement was observed in three of these patients, with a response time ranging from 4 to 8 days. At 90 days, two patients achieved steroid-free clinical and endoscopic remission and one showed clinical response, while the remaining patient underwent total colectomy [83].

In a different case series, six steroid-refractory patients with ASUC previously exposed to Infliximab received Upadacitinib 45 mg. Clinical response was induced by Upadacitinib in all six patients during hospitalization, four of them achieving corticosteroid-free clinical remission at 8 weeks with complete resolution of rectal bleeding. At week 16, sustained clinical remission was observed in five out of these six patients, while one patient underwent colectomy for refractory disease [84].

In another case series including twelve patients (seven UC, three colonic CD and two unclassified chronic colitis), they were treated with Upadacitinib. Overall, four patients required surgery, one patient did not discontinue steroids ≤ 3 months after hospital discharge and one patient required re-induction with Upadacitinib. Clinical remission ≤ 90 days was observed in five (63%) patients [85].

## 9. Expert Opinions

Several new treatments have recently been developed for treating patients with active UC. Nevertheless, clinicians still have to face difficulties in managing patients showing refractoriness or intolerance to these drugs. Moreover, in UC, the therapeutic ceiling currently represents a major issue in IBD. Due to these limitations of currently available drugs, subgroups of patients with UC fail to reach long-term steroid-free remission. Thus, the development of the new small molecules, and in particular of the JAK-i, may help to address this issue.

JAK-i show multiple upsides when compared to previously available therapies for UC, including the rapidity of onset and the low immunogenicity. Moreover, a satisfactory safety profile of these newly developed small molecules has been reported. Furthermore, one of the most relevant characteristics of these drugs is the oral route of administration, which may lead to better patient compliance and tolerability.

All JAK-i show a rapid onset of action, determining, in subgroups of patients, a reduction in rectal bleeding within a few days. Clinical efficacy, rather than the capacity of inducing endoscopic and histological healing, currently appears as one of the main characteristics of JAK-i. MH can indeed be achieved in patients on JAK-i, although in a relatively low percentage of patients.

Safety has initially been a major issue when considering the use of JAK-i in UC. The first of these drugs used for UC, Tofacitinib, was indeed burdened by the association with a higher risk of VTE. This AE was already reported in patients with other conditions, as a non-inferiority study comparing Tofacitinib and anti-TNFα reported a higher risk of thromboembolism and of major adverse cardiovascular events (MACEs) using this small molecule [86]. The development of selective JAK-i strongly mitigated this limitation, as thromboembolic events and MACEs were not reported as a major concern, according to both clinical trials and real-life studies. The most frequent and relevant AEs linked to the use of selective JAK-i are currently represented by infections, particularly due to HZV. Differently, safety is still a major concern in case of pregnancy and lactation, as preclinical data suggest a possible risk of malformation related to inhibition of the JAK-STAT signaling.

Among the main limitations of the present review, the reported findings do not derive from a systematic revision of the use of small molecules in patients with UC. Moreover, only the main results from all the reported trials using JAK-i in UC have been summarized. Finally, only the most relevant comparisons between UC and other conditions (i.e., rheumatoid arthritis) in terms of efficacy and safety of these molecules have been reported.

Most of the new small molecules for treating moderate-to-severe UC currently show, in appropriately selected subgroups of patients, a satisfactory safety profile. Their modality of administration coupled with the novel mechanism of action make these drugs an important improvement in the therapeutic armamentarium for treating patients with UC. Indeed, recent evidence has confirmed the efficacy of these drugs and particularly Upadacitinib for moderately to severely active UC, even when used as first-line treatment [87,88]. Even data from a recent network meta-analysis suggested that in biologic/JAK-I naïve patients, Upadacitinib is associated with significant improvements versus most other second- and third-line emerging therapies [89]. Due to the potential AEs related to the use of these treatments, indication needs to be given by experienced IBD-dedicated gastroenterologists, as for all the recently introduced immunomodulatory drugs.

## 10. Future Directions

Optimization of the currently available JAK-i represents one of the main issue when considering the use of these treatments in UC. This is in terms of selection of responsive patents, type of JAK-i and concomitant treatments. Nevertheless, the development and use of new JAK-i may also add new insight for proper treatments of patients with UC. In the changing landscape of advanced therapies for UC, the development and implementation of these therapies is expanding the range of medical options to treat UC. However, further long-term data, real-world studies and head-to-head trials are warranted to better define the optimal sequencing of treatments for patients with UC [90].

In this regard, phase 2b/3 clinical trials in UC have been made on three additional small molecules acting as JAK-i, represented by Ritlecitinib, Brepocitinib and Izencitinib. Ritlecitinib is a highly selective inhibitor of JAK3 and the TEC kinase family. Brepocitinib is a dual inhibitor that combines the anti-inflammatory effects by inhibiting both JAK1- and TYK2-mediated IL-12 and IL-23 signaling [91,92]. These drugs have been tested in a phase 2b, parallel-arm, double-blind umbrella study performed on 317 patients with UC. The reported clinical outcomes of clinical remission at week 8 were 32.7% and 25.5%, respectively, for the higher doses in Ritlecitinib and Brepocitinib groups [93].

Izencitinib (TD-1473) was designed as an orally administered, gut-selective, pan-JAK-i aimed to minimize systemic exposure [94]. Available data report that the study drug failed to meet the primary efficacy end-point of change in the total Mayo score at week 8 in patients with moderate-to-severe UC [95].

Alongside this, great efforts are still required in order to develop new safe and effective drugs and to identify predictors of response, aimed to allow personalized treatments in patients with UC.

## 11. Conclusions

During the last few years, JAK-i have been added to previous treatments for UC, and IBD-dedicated clinicians are still learning how to optimize their use according to specific characteristics. Growing data are indeed becoming available on the efficacy of JAK-i in many different situations, from ASUC to chronically active disease, frequently with reassuring results.

Considering their mechanism of action, efficacy rate and safety profile, the use of small molecules in UC is promising. Appropriate selection of the subgroups of patients with UC suitable for a safe treatment with JAK-i is mandatory in order to avoid potential adverse events related to these molecules. Further growth of our knowledge regarding predictive markers of response to JAK-i coupled with optimization of their use (monotherapy or combined with other immunomodulators) are required. The achievement of these targets may lead to better disease control in a large proportion of patients with UC.

## Figures and Tables

**Table 1:** Clinical trials of currently approved Janus kinase inhibitors for Ulcerative Colitis.

Drug	Dose	Trial	Patients (*n*=)	Efficacy	Mucosal Healing	Adverse Events	Serious Adverse Events
**Tofacitinib**	Induction therapy: 10 mg t.i.d. vs. placebo for 8 weeks	OCTAVE Induction 1: phase 3, randomized, double-blind, placebo-controlled trial	598	Clinical remission (vs. placebo): 18.5% vs. 8.2% (*p* = 0.007)	MH at 8 weeks (vs. placebo): 31.3% vs. 15.6% (*p* < 0.001)	Tofacitinib (56.5%) Placebo (59.8%)	Tofacitinib (3.4%) Placebo (4.1%)
**Tofacitinib**	Induction therapy: 10 mg t.i.d. vs. placebo for 8 weeks	OCTAVE Induction 2: phase 3, randomized, double-blind, placebo-controlled trial	541	Clinical remission (vs. placebo): 16.6% vs. 3.6% (*p* < 0.001)	MH at 8 weeks (vs. placebo): 28.4% vs. 11.6% (*p* < 0.001)	Tofacitinib (54.1%) Placebo (52.7%)	Tofacitinib (4.2%) Placebo (8.0%)
**Tofacitinib**	Maintenance therapy: 5 mg or 10 mg t.i.d. vs. placebo for 52 weeks	OCTAVE Sustain: phase 3, randomized, double-blind, placebo-controlled trial	593	Clinical remission (5 mg, 10 mg vs. placebo): 34.3% vs. 40.6% vs. 11.1% (*p* < 0.001 vs. placebo)	MH at 52 weeks (5 mg,10 mg vs. placebo): 37.4% vs. 45.7% vs. 13.1% (*p* < 0.001 vs. placebo)	Tofacitinib 10 mg (79.6%) 5 mg (72.2%) Placebo (75.3%)	Tofacitinib 10 mg (5.6%) 5 mg (5.1%) Placebo (6.6%)
**Upadacitinib**	Induction therapy: 45 mg o.d. vs. placebo for 8 weeks	UC1: phase 3, multicenter, double-blind, randomized trial	474	Clinical remission (vs. placebo): 26% vs. 5% (*p* < 0.0001)	MH at 8 weeks (vs. placebo): 11% vs. 1% (*p* < 0.0001)	Upadacitinib (56%) Placebo (62%)	Upadacitinib (3%) Placebo (6%)
**Upadacitinib**	Induction therapy: 45 mg o.d. vs. placebo for 8 weeks	UC2: phase 3, multicenter, double-blind, randomized trial	522	Clinical remission (vs. placebo): 34% vs. 4% (*p* < 0.0001)	MH (vs. placebo): 13% vs. 2% (*p* < 0.0001)	Upadacitinib (53%) Placebo (40%)	Upadacitinib (3%) Placebo (5%)
**Upadacitinib**	Maintenance therapy: 15 mg o.d. vs. 30 mg o.d. vs. placebo for 52 weeks	UC3: phase 3, multicenter, double-blind, randomized trial	451	Clinical remission (15 mg, 30 mg vs. placebo): 42% vs. 52% vs. 12% (*p* < 0.0001 vs. placebo)	MH (15 mg, 30 mg vs. placebo): 18% vs. 19% vs. 5% (*p* = 0.0003; *p* = 0.0001)	Upadacitinib 30 mg (79%) Upadacitinib 15 mg (78%) Placebo (76%)	Upadacitinib 30 mg (6%) Upadacitinib 15 mg (7%) Placebo (13%)
**Filgotinib**	Induction study and maintenance studies: Filgotinib 200 mg vs. Filgotinib 100 mg vs. placebo o.d.	SELECTION: phase 2b/3, double-blind, randomized, placebo-controlled trial	659 inductionstudy A	Clinical remission at week 10: Study A (100 mg, 200 mg vs. placebo): 19.1% vs. 26.1% vs. 15.3% (*p* = 0.34 vs. *p* = 0.0157)	MH Induction study A (100 mg, 200 mg vs. placebo): 5.8% vs. 3.6% (*p* = 0.34) 12.2% vs. 3.6%, (*p* = 0.0047)	Filgotinib 200 mg (53.6%) Filgotinib 100 mg (50.4%) Placebo (56.3%)	Filgotinib 200 mg (4.3%) Filgotinib 100 mg (5%) Placebo (4.7%)
			689 induction study B	Study B (100 mg, 200 mg vs. placebo): 9.5% vs. 11.5% vs. 4.2% (*p* = 0.06 vs. *p* = 0.013)	MH Induction study B (100 mg, 200 mg vs. placebo): 2.1% vs. 2.1% (*p* = 0.99) 3.4% vs. 2.1%, (*p* = 0.42)		
			664 maintenance study (391 from study A, 273 from study B)	Clinical remission at week 58 (100 mg, 200 mg vs. placebo): 23.8% vs. 13.5% (*p* = 0.04) 37.2% vs. 11.2%, (*p* < 0.0001)	Maintenance study (100 mg, 200 mg vs. placebo): 13.4% vs. 7.9% (*p* = 0.18) 15.6% vs. 6.1%, (*p* = 0.0157)	Placebo in responders to: Placebo (61.3%) Filgotinib 100 mg (65.9%) Filgotinib 200 mg (59.6%) Filgotinib 100 mg (60.3%) 200 mg (66.8%)	Placebo in responders to: Placebo (4.3%) Filgotinib 100 mg (7.7%) Filgotinib 200 mg (0%) Filgotinib 100 mg (4.5%) 200 mg (4.5%)

Abbreviations: MH = mucosal healing; vs. = versus.
